# Rapid progression of traumatic bifrontal contusions to transtentorial herniation: A case report

**DOI:** 10.1186/1757-1626-1-203

**Published:** 2008-10-02

**Authors:** Tausif Rehman, Rushna Ali, Isaac Tawil, Howard Yonas

**Affiliations:** 1 MSC10 5615, Department of Neurosurgery, University of New Mexico, Albuquerque, NM, USA; 2 School of Medicine, Aga Khan University, P.O Box 3500, Stadium Road, Karachi, Pakistan

## Abstract

We report a case of mild to moderate traumatic brain injury in which ICP monitoring or quantitative cerebral perfusion data may have allowed earlier recognition of impending herniation, avoidance of a secondary insult, and ultimately resulted in a better outcome, even though the patient did not meet the standard guidelines of the Brain Trauma Foundation. A thirty-five year old male who presented with traumatic bifrontal contusions and GCS of fourteen and twelve hours later progressed rapidly to having dilated pupils and transtentorial/central herniation over the course of fifteen minutes. The patient was taken emergently for a bifrontal craniectomy. Post operatively he had an acute infarct in the posterolateral left temporal lobe with expected evolution of parenchymal contusions as well as infarcts in the splenium of the corpus callosum, left thalamus and medial right occipital lobe. This case signifies an exception from the guidelines submitted by the Brain Trauma Foundation for intracranial pressure monitoring in patients with severe brain injury.

We also point out previous reports which state that in such a patient a more sensitive test for detection would perhaps be quantitative blood flow monitoring, and may have led to a better outcome. We recommend using intracranial pressure monitoring or blood flow measurements to trend patients with bifrontal intraparenchymal contusions and GCS greater than eight to prevent clinically undetected deterioration from transtentorial/central herniation.

## Background

As per the guidelines of the brain trauma foundation, intracranial pressure (ICP) monitoring is recommended for patients with severe head injury which is defined as a Glasgow Coma Scale (GCS) of 3–8 after cardiopulmonary resuscitation, with an abnormal admission computed tomography (CT) scan. An abnormal CT scan is defined as one revealing hematomas, contusions, edema or compressed basal cisterns. ICP monitoring is also deemed appropriate for patients with severe head injury with normal CT scans if they meet two or more of the following criteria: age over forty years, unilateral or bilateral motor posturing, or systolic blood pressure <90 mmHg [1].

In patients with mild to moderate head injury (GCS 9–15) ICP monitoring is not routinely recommended as fewer than twenty percent of these patients progress to severe brain injury with intracranial hypertension. However, the guidelines allow for physician selection of certain conscious patients who may benefit from ICP monitoring.

We report a case of mild to moderate traumatic brain injury in which ICP monitoring or quantitative cerebral perfusion data may have allowed earlier recognition of impending herniation, avoidance of a secondary insult, and ultimately resulted in a better outcome.

## Case presentation

A thirty five year old male suffered a fall from approximately ten feet at a construction site, reportedly striking his head on the ground. The patient subsequently had a witnessed tonic/clonic seizure lasting approximately thirty seconds. Paramedics had initially reported a Glasgow Coma Score of six. The patient had no known comorbids and was not on any medication.

**Figure 1:**
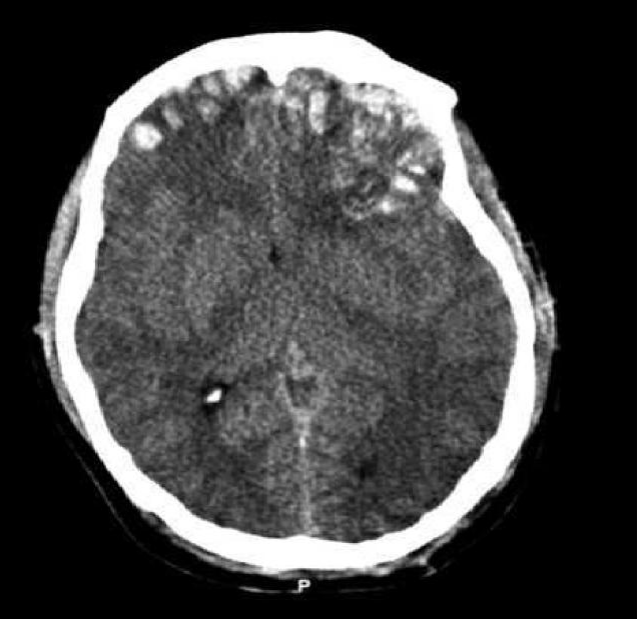
(Initial CT.tif) – Preoperative CT scan of patient while he had a GCS of 14.

The patient's emergency department course was not complicated by hypotension or hypoxia. In the emergency department the patient's GCS score was ten. Emergent CT imaging revealed a sagittally oriented skull fracture extending from the vertex to the foramen magnum as well as a transverse parietal and temporal bone fracture. Multiple frontal, parietal, and temporal lobe contusions with associated interhemispheric hemorrhage and a left-sided subdural hematoma measuring 1.7 mm in greatest depth were appreciated. Effacement of the basilar cisterns was noted without shift of midline structures. (Figure 1)

The patient subsequently was admitted to the neurosurgical intensive care unit for close observation. The initial exam upon admission to the ICU revealed a GCS of ten (E4, M5, V1). Coagulation parameters were within normal limits. The decision was made to proceed without ICP monitoring. The patient received a loading dose of Fosphenytoin, maintenance intravenous fluids and insulin as needed for tight glycemic control. No sedation was administered.

The patient's clinical status during the following fourteen hours improved significantly to a GCS of 14. The patient began to follow simple commands. A repeat CT scan six hours after admission showed interval evolution of the contusions with pericontusional edema in the frontal, parietal and temporal lobes bilaterally. Persistent effacement of the basilar cisterns was appreciated. The subdural hematoma did not increase in size and again there was no shift of midline structures.

Approximately 15 hours after admission, the patient deteriorated over several minutes. His mental status declined, first losing the ability to follow commands and rapidly becoming unresponsive with fixed and dilated pupils. The CT scan at that time showed no significant change in the volume of the hemorrhagic contusions or position of midline structures. The patient was then taken directly to the operating room for a bilateral frontal craniectomy and evacuation of contusions. In the operating suite the patient's dura was found to be under tension. The dura was opened and contusions decompressed bilaterally. The patient's falx cerebri was also dissected allowing decompression and greater anteriorsuperior movement of the brain.

**Figure 2:**
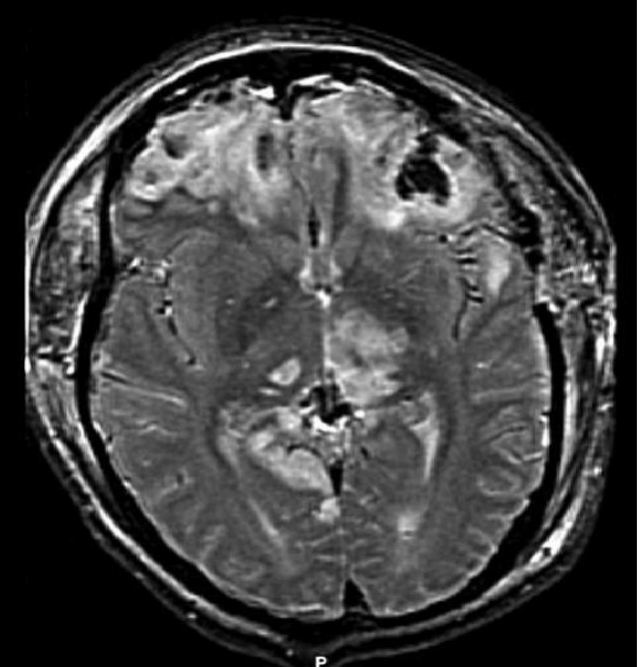
(MRI.tif) – Postoperative MRI showing evidence of injury due to herniation.

The intraparenchymal pressure monitor then placed in the right frontal lobe revealed ICP ranging between seven and ten mm Hg. The patient's pupillary function improved post-operatively to two millimeters and they were equal and reactive. Supportive care continued in the intensive care unit. The repeat CT scan eight hours later revealed new infarcts in the left thalamus and right occipital lobe. Post-operative magnetic resonance imaging (MRI) was performed on admission day five that confirmed an acute infarct in the posterolateral left temporal lobe with expected evolution of parenchymal contusions as well as infarcts in the splenium of the corpus callosum, thalami (left thalamus) and medial right occipital lobe. (Figure 2)

At one month from admission the patient made moderate functional improvements; following commands intermittently and speaking slowly. He has residual right hemiparesis while the left upper extremity strength is near normal.

## Discussion

This case demonstrates a patient who rapidly deteriorated after suffering from mild to moderate traumatic brain injury due to central herniation after an improving and relatively high GCS. The infarctions involving the thalamus, medial temporal lobes and occipital lobe are evidence of cephalo-caudal migration of the basal ganglia and adjacent structures. Central herniation caused attenuation of the posterior cerebral artery and the thalamoperforating vessels and subsequent stroke.

Historically, this process has been associated with rapid clinical deterioration from mild neurologic dysfunction to lethal mid-brain compression. While it is generally believed that head injury patients with GCS 9–15 are at low risk for intracranial hypertension, the limitations of the Glasgow Coma Scoring for predicting clinical deterioration are well recognized. In particular, the deficits produced by frontal lobe damage including memory loss, behavioral changes and poor executive function are not accounted for in the GCS.

The question then is to establish what additional information may have led to earlier recognition of impending herniation. We postulate that an ICP monitor may have revealed worsening pressure trends prior to discovery of signs of impending herniation. An intraventricular catheter would have been a useful therapeutic temporizing measure until surgical decompression was performed. However, as reported by Wozney [2], a patient with a similar pattern of injury suffered transtentorial herniation while using ICP monitoring plus Xenon enhanced CT blood flow data. In this case, ICP values were unchanged while Xenon CT revealed decreasing blood flow in the frontal lobe and central ganglionic structures.

Current literature supports bifrontal decompressive craniectomy as a treatment option for patients with diffuse, intractable post traumatic cerebral edema with resultant intra-cranial hypertension and GCS of 6–8 [3]. However, our patient would not be included using these criteria. Identifying the subset of patients with mild to moderate brain injury who would benefit from ICP monitoring is a difficult task, given the morbidity associated with invasive monitors and the small numbers of these patients who incur intracranial hypertension [1]. A less invasive predictor of our patient's clinical course would have beenquantitative blood flow determination by Xenon enhanced CT. Thus, we recommend using blood flow measurements to trend patients with bifrontal intraparenchymal contusions and GCS greater than eight to prevent clinically undetected deterioration from central herniation.

## Competing interests

The authors declare that they have no competing interests.

## Authors' contributions

All the above mentioned authors have contributed significantly to this case report. TR was involved in the operative and post-operative care of this patient, and contributed significantly to the preparation of the manuscript. RA was involved in gathering the relevant data and manuscript writing. IT was incharge of the post-operative ICU care and decision making process for this patient. HY was the attending neurosurgeon responsible for the entire course of in-hospital care for this patient and in putting the manuscript together. All authors read and approved the final manuscript.

## Consent

Written informed consent was obtained from the patient for publication of this case report and accompanying images. A copy of the written consent is available for review by the Editor-in-Chief of this journal.
